# The epidemiology of malaria in adults in a rural area of southern Mozambique

**DOI:** 10.1186/1475-2875-6-3

**Published:** 2007-01-17

**Authors:** Alfredo Mayor, John J Aponte, Carole Fogg, Francisco Saúte, Brian Greenwood, Martinho Dgedge, Clara Menendez, Pedro L Alonso

**Affiliations:** 1 Center for International Health, Hospital Clinic, IDIBAPS, University of Barcelona, C/Rosello, 132, 2° 2^a^, 08036 Barcelona, Spain; 2 Manhiça Health Research Center, Manhiça, Mozambique; 3 London School of Hygiene and Tropical Medicine, London, UK; 4 National Malaria Control Programme, Ministry of Health, Mozambique

## Abstract

**Background:**

Epidemiological studies of malaria in adults who live in malaria endemic areas are scarce. More attention to the natural history of malaria affecting adults is needed to understand the dynamics of malaria infection and its interaction with the immune system. The present study was undertaken to investigate the clinical, parasitological and haematological status of adults exposed to malaria, and to characterize parasites in these individuals who progressively acquire protective immunity.

**Methods:**

A cross-sectional survey of 249 adults was conducted in a malaria endemic area of Mozambique. Clinical, parasitological and haematological status of the study population was recorded. Sub-microscopic infections and multiplicity of infections were investigated using polymerase chain reaction (PCR) and restriction fragment length polymorphism of *Plasmodium falciparum *merozoite surface protein 2 (*msp2*).

**Results:**

Prevalence of *P. falciparum *infection by microscopy (14%) and PCR (42%) decreased progressively during adulthood, in parallel with an increase in the prevalence of sub-microscopic infections. Anaemia was only related to parasitaemia as detected by PCR. Multiplicity of infection decreased with age and was higher in subjects with high *P. falciparum *densities, highlighting density-dependent constraints upon the PCR technique.

**Conclusion:**

Adults of Manhiça progressively develop non-sterile, protective immunity against *P. falciparum *malaria. The method of parasite detection has a significant effect on the observed natural history of malaria infections. A more sensitive definition of malaria in adults should be formulated, considering symptoms such as diarrhoea, shivering and headache, combined with the presence of parasitaemia.

## Background

The epidemiology of malaria in adults who live in malaria endemic areas is a neglected area of research. Malaria control strategies have focussed on children under the age of 5 years and pregnant women, as the majority of malaria-related sickness and death is seen in these two groups [1]. However, early studies in West Africa showed that clinical attacks of malaria also occur in adults living in areas of high endemicity [2] and a recent report points out the considerable contribution of malaria as a cause of death in adults [3].

The risk of malaria attacks in residents of malaria endemic areas falls as they become older [4], suggesting that protection is a function of age. This protective immunity is sequentially being reflected first by a reduction of life-threatening disease, then by a fall in the incidence of mild malaria and finally by a reduction in parasite prevalence [5]. The means by which acquired immunity develops is still a matter of contention. It is likely that cumulative exposure to the enormous repertoire of antigenic variants of blood stage malaria parasites [6] plays an important part. However, there is evidence which suggests that protection can be established after a relatively brief period of exposure and lasts for many years [7,8]. Indeed, passive transfer of naturally acquired immunity [9] and malariotherapy of syphilitic patients [10] have suggested that there is a strong component of non-variant specific immunity involved in the protection against malaria which may be age-dependent.

The immune mechanisms that deal with a malarial infection probably change with age, as suggested by the age-dependency of malariometric indices [11], the pyrogenic threshold of parasitaemia [12], the speed with which infections are controlled, the incidence of clinical episodes [7] and the parasitological complexity of individual infections [13]. It is generally accepted that protective immunity effectively prevents the severe clinical manifestations of *Plasmodium falciparum *infections and substantially reduces parasite loads, but does not prevent infection [14]. The consequence of this process is that the presence of blood-stage parasites in a semi-immune host is not synonymous with disease. This, together with the non-specificity of the malaria signs and symptoms in adults, make the individual diagnosis of clinical malaria in adults difficult in highly endemic areas.

Studies of malaria in semi-immune adults of Africa are scarce [12,15-19]. More attention to the natural history of malaria affecting adults is needed to understand the dynamics of malaria infection and its interaction with the immune system. The present study was undertaken to investigate the clinical, parasitological and haematological status of adults living in a region in Mozambique where malaria is endemic, and to characterize parasites in these individuals who progressively acquire protective immunity.

## Methods

### Study area

The study took place at the Manhiça Health Research Center, in Manhiça (Maputo Province), a rural area in Southern Mozambique. The area, described in detail elsewhere [20], is covered by a continuous demographic surveillance system (DSS) run by CISM since 1996. Malaria transmission is continuous, with intense seasonality. The entomological inoculation rate at the time of the study was 15 infective bites/person/year [21]. *Anopheles funestus *is the main vector and most infections are caused by *P. falciparum*.

### Cross sectional survey

A sample of 500 adults aged ≥ 15 years was selected randomly from the 1999 demographic census. Potential participants were invited to attend selected sites on a given date during the period 26^th ^July to the 4^th ^August 1999. Pregnant women and individuals who had taken anti-malarial treatment in the week preceding sampling were excluded. After written and witnessed informed consent had been obtained, capillary blood samples were collected by finger prick for preparation of thick and thin blood films, haematocrit and filter paper blot samples (Schleicher & Schuell number 903™ filter paper, Dassel, Germany). Axillary temperature and details of symptoms potentially due to malaria (reported fever, headache, body pain, shivering, nausea, vomiting, abdominal pain and diarrhoea) were recorded. Results of blood film examination and the haematocrit were sent to participants within two days of sampling, and medical advice and treatment were given when necessary.

### Laboratory methods

Blood slides were processed and read according to quality-controlled procedures as described elsewhere [22]. Haematocrit values were determined in a microcapillary after centrifugation for 5 min at 9000 g. All samples positive by light microscopy, those with a haematocrit less than 33%, and 30% of the remaining negative slides, were analysed by polymerase chain reaction (PCR) for *msp2 *and restriction-fragment length polymorphism (RFLP). A piece corresponding to approximately 1/8^th ^of the blot on the filter paper was used for a nested PCR amplification of *msp2 *and genotyping by restriction with enzymes as described elsewhere [23]. A negative control, to which no DNA had been added, and a positive control, to which *P. falciparum *clone 3D7 DNA (50 ng) had been added were included in each set of amplification reactions.

### Data analysis

Data were double entered using Visual Fox Pro 5.0 and statistical analyses were performed using Stata5. Fever was defined as an observed axillary temperature ≥37.5°C and/or patient-reported fever within the previous 24 h. Clinical malaria was defined as fever with a positive blood slide for asexual *P. falciparum *parasites. Anaemia was defined as a haematocrit <33%. The summary measures consisted of percentage for categorical variables and means for continuous variables. Parasite densities are reported as the geometric mean of parasite-positive samples. The prevalence of sub-microscopic infections was calculated as the number of the PCR-positive samples which had been negative by microscopic examination divided by the total number of PCR-positive samples. Multiplicity of infection (MOI) was defined as the number of distinct *msp2*-genotypes detected in infected samples. Student's t-test and linear regression were used to assess differences between quantitative variables. The age trends of the different categorical variables were assessed using the Chi-square test for linear trend. The association between parasite density and MOI was calculated separately for each age group by multiple regression analysis. Categorical variables such as sex, positivity by PCR and microscopy, anaemia and presence or absence of symptoms were analysed using Chi-square and logistic regression. A p-value of 0.05 was considered statistically significant. Sensitivity and specificity of the malaria case definition was evaluated using the attributable fraction of fever due to malaria as described elsewhere [24].

### Ethical clearance

This study fell within the national clearance granted in 1996 to the malaria epidemiology studies of the Manhiça Health Research Center by the Ministry of Health/National Institute of Health of Mozambique.

## Results

Among the 500 names selected from the census, 137 were not traceable. Eleven of the remainder had taken antimalarials, 12 were pregnant and 91 were absent on the day of the survey. Thus, the study included 249 adults whose mean age was 37 years (range 15 to 83 years). In concordance with the overall sex distribution of the adult population in the area, two thirds of the subjects were female.

**Figure 1:**
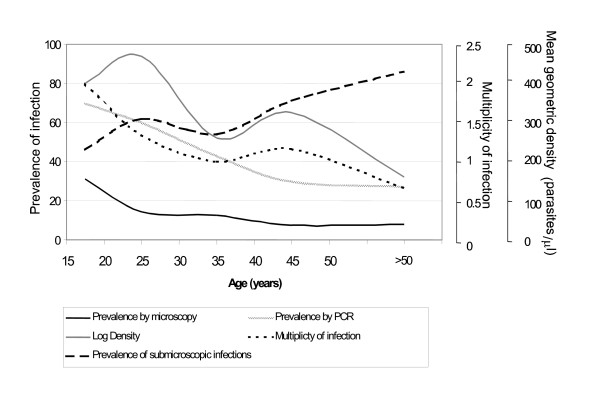
Prevalence of *Plasmodium falciparum *infection as determined by microscopy or PCR, proportion of sub-microscopic infections, mean geometric density and multiplicity of infection by age in adults from Manhiça.

Parasitaemia as detected by microscopy was found in 14.4% of subjects (36/249). All infections were due to *P. falciparum*. Only one subject had gametocytes. Prevalence of parasitaemia by microscopy decreased significantly with increasing age (p < 0.001) (Figure 1). The geometric mean parasite density was 336 parasites/μl (range 32 to 20376 parasites/μl), and also decreased with age, but this relation was not significant (p = 0.31) (Figure 1).

A total of 180 samples, selected as defined above, were analysed by PCR. The prevalence of *P. falciparum *infection determined by PCR was 42%, and significantly decreased with age (p < 0.001) (Figure 1). The proportion of sub-microscopic infections increased significantly with age (p = 0.02) (Figure 1). A small number (8.3%) (3/36) of the parasite positive samples by microscopy were negative by PCR while 34.7% (50/144) of the samples negative by light microscopy were positive by PCR. The specificity of light microscopy was 96.9% but the sensitivity only 37.5%.

All PCR-positive samples were analysed for MOI by RFLP. Of these 50.6% (41/81) consisted of a single *msp2 *genotype, 23.5% were double infections, 14.8% triple, 9.9% quadruple, and a quintuple infection. The mean MOI was 1.9. There was an inverse association between age and MOI (p = 0.009) (Figure 1). Doubling the age, reduced the MOI by 20.0%. Subjects with microscopically detectable parasites were more likely to have multiple infections than those who were just PCR positive (p = 0.010). After adjusting for age, there was a positive association between parasite density and MOI (p = 0.001). Every new infection increased the mean geometric parasite density by a factor of 1.98.

The mean axillary temperature was 36.1°C (range 35.1°C to 37.3°C). None of the adults had fever at the moment of the examination. No significant associations were found between mean temperatures and the prevalence of any symptom, but a significant inverse relationship was found between age and temperature. Doubling age decreased temperature by 0.05°C (p = 0.004). Prevalences of symptoms are shown in Table 1.

**Table 1:** Prevalence of symptoms and signs in the study population (n = 249).

**SYMPTOM/SIGN**	**Prevalence (%)**
***Individual symptoms***	
Reported fever	15.7
Headache	23.3
Body pain	14.1
Shivering	12.1
Nausea	12.9
Vomiting	5.6
Abdominal pain	37.4
Diarrhoea	6.8
***Number of symptoms experienced***	
No symptoms	48.6
One symptom	24.9
2/3 symptoms	12.5
4+ symptoms	14.1
***Signs***	
Fever	0

Anaemia (haematocrit < 33%) was found in 9.8% (24/245) of the subjects (in 4 the haematocrit could not be measured). Twenty-two of the 24 anaemic subjects were females. Haematocrit values were not related to age (p = 0.5). After adjusting for sex, PCR positive subjects had a haematocrit 2.0% lower than the PCR negative subjects (p = 0.002).

Clinical malaria, as defined above, was found in 2.4% of the subjects (6/249). The estimated attributable fraction of fever due to malaria was 6.7%. The specificity of our definition was estimated as 91% (95% CI 81% to 98%). Among parasitaemic adults, there was a significant association between high parasite densities and the presence of diarrhoea (p = 0.004) or headache (p = 0.014), and an inverse association between shivers and parasite density (p = 0.029). No relationship was found between MOI and any symptom.

## Discussion

This study, one of the few focussing on malaria in adults from a malaria hyperendemic area [7,12,15-19], has shown a high discordance between *P. falciparum *prevalence as determined by microscopy (14%) and PCR (42%). Estimates of infection determined by microscopy are probably unreliable in adults because of the high prevalence of *P. falciparum *infections with densities below the detection threshold of blood film examination. Even prevalence of PCR detected parasites and MOI may be underestimated when parasites reach such low levels that densities fluctuate around the threshold limit of the PCR [25]. This interpretation is supported by the density-dependent constraints of the PCR found in our study; multiple *P. falciparum *clones are differentiated more efficiently in high-density infections.

The abundance of sub-microscopic infections in adults from Manhiça and the low specificity of signs and symptoms hamper the definition of a malaria episode. Fever was not detected at the time of examination in any of the adults in this study. Other symptoms such as diarrhoea, shivering and headache, combined with the presence of parasitaemia, could be used to formulate a more sensitive definition of malaria in adults. Special emphasis should be paid to anaemia. In our study area, the significantly higher frequency of anaemia cases among PCR positive individuals might have been due to a reduction in haematocrit because of recent high density infections that have been controlled at the moment of the examination by an efficient immunity or prior treatment. Alternatively, it may have been the result of persistent asymptomatic infections that significantly increase the risk of becoming anaemic.

During the dry season, almost half of the adults from Manhiça were infected by *P. falciparum*, as detected by PCR. Parasite rate might be even higher during the wet season. Studies in other settings have found similar high prevalences [25], showing that cumulative prevalence of parasite carriers over a year is close to 100% [26]. This prevalent carriage of parasites can be due to a chronic nature of malaria infections, or to frequent reinfections which only reach low blood-stage densities before being eliminated. Most of the infections in our study subjects were asymptomatic, indicating the occurrence of host immunity able to restrict parasites to low densities with a sub-clinical course, although these immune mechanisms cannot achieve permanent sterility. Importantly, immunity seems to develop during the whole life, as suggested by the continuous increment in the prevalence of sub-microscopic infections and the trend towards a reduction of parasite densities, which may not reach statistical significance because of the impossibility to assign accurate densities to sub-microscopic infections by conventional PCR methods.

Two forms of antimalarial immunity are believed to occur, an anti-disease and an anti-parasite immunity [27]. The first one, reflected by the age evolution of fever, is believed to develop rapidly, reducing the frequency of clinical presentation of malaria. The second is said to be slowly acquired, leading to a progressive decrease in parasite prevalence and MOI. However, the immune ability to control the parasite may be better reflected by parasite densities rather than by the prevalence of parasites. We have reported previously that in the Manhiça district of Mozambique, both parasite densities and incidence of clinical malaria decline in parallel during the second half of infancy [28,29], suggesting that immune mechanisms able to control parasite densities result in less severe symptoms. In this community anti-disease and anti-parasite immunity seem to develop concomitantly and do not necessarily reflect two different aspects of the host defence mechanisms against malaria.

## Conclusion

This study has evidenced the abundance of sub-microscopic infections in adults from Manhiça. Estimates of infection determined by microscopy are probably unreliable, suggesting that the method of parasite detection may have a significant effect on the observed natural history of malaria infections in adults. The increment with age in the prevalence of sub-microscopic infections, together with the trend towards a reduction of parasite densities, suggests that immunity in adults develops during the whole life. This immunity is able to restrict *P. falciparum *parasites to low densities with a sub-clinical course, but is not sterilizing. A more sensitive definition of malaria in adults should be formulated, considering other symptoms such as diarrhoea, shivering and headache, combined with the presence of parasitaemia.

## Authors' contributions

AM carried out the molecular genotyping study, the analysis and interpretation of data and prepared the manuscript. JA carried the statistical analysis and helped to draft the manuscript. CF carried out the sample collection and contributed to the analysis and interpretation of data. FS, BG and MD contributed to the interpretation of the data and drafting of the paper. CM and PA conceived and coordinated the study, participated in the analysis and interpretation of the data, and contributed to the preparation of the manuscript. All authors read and approved the final manuscript.
